# Relapse of rare diseases during COVID-19 pandemic: bicytopenia in an adult patient with thiamine-responsive megaloblastic anaemia

**DOI:** 10.11604/pamj.supp.2020.35.139.25368

**Published:** 2020-08-10

**Authors:** Nourelhouda Nouira, Rawdha Mansouri, Rami Tlili, Ines Bhouri, Souha Sfaxi, Dorra Chtourou, Maamoun Ben cheikh

**Affiliations:** 1 Mongi Slim Academic Hospital, Emergency Department, Faculty of Medicine of Tunis, University of Tunis El Manar, Tunisia,; 2 Aziza Othmana Academic Hospital, Hematology Department, Tunisia,; 3 Cardiology Department, Mongi Slim Academic Hospital, Tunisia

**Keywords:** Thiamine, megaloblastic anaemia, diabetes, genetics, COVID-19 pandemic

## Abstract

Thiamine-responsive megaloblastic anaemia (TRMA) is a syndrome associated with megaloblastic anaemia, diabetes mellitus and sensorineural deafness, due to mutations in the SLC19A2gene, which codes for a thiamine carrier protein. Oral thiamine supplementation is the main treatment. We report the case of a 19-year-old man known for TRMA, who presented in the emergency department with bicytopenia (haemoglobin 5,4 g/dL, thrombocytes 38×10^9^/L) revealed by dyspnea and chest pain. Investigations excluded bleeding, hemolysis, coagulopathy and iron deficiencies. A recent infection and an acute coronary syndrome have also been eliminated. We later found out that thiamine treatment had been discontinued three months before, due to general confinement in Tunisia during the COVID-19 pandemic. Parenteral administration of 100 mg of thiamine daily resulted in the recovery of haematopoiesis within three weeks.

## Introduction

In the context of COVID-19 pandemic, with the obligation of containment and social distancing, patients suffering from rare syndromes may have difficulty to maintain their medical monitoring and their treatment. Among these rare diseases, the Thiamine-responsive megaloblastic anemia syndrome (TRMA) which is an autosomal recessive disorder with features that include megaloblastic anemia, sensorineural deafness and diabetes mellitus. In this disease, the active thiamine absorption into cells is disturbed. Treatment with pharmacological doses of thiamine ameliorates the megaloblastic anemia and diabetes mellitus. This supplementation must be continued for life otherwise relapse of anemia or even a bi or pancytopenia is inevitable. We present a case of bicytopenia in a young adult with known TRMA.

## Patient and observation

**Case presentation:** a 19-year-old man known for TRMA, who presented in the emergency department because of progressively worsening dyspnea, chest pain and paleness. He was born in Tunisia to non-consanguineous Tunisian parents. At the age of 12 months, diagnosed megaloblastic anaemia, insulin-dependent diabetes mellitus and severe bilateral sensorineural hearing raised suspicion of TRMA. Thiamine treatment (300 mg orally daily) led to normalization of hemoglobin level. Permanent insulin treatment with three fixed doses per day was necessary because of elevated glucose levels. The patient had consulted our emergency service on July, 2020. We found the notion of stopping treatment for three months since the beginning of the COVID-19 pandemic. Our patient lives in a rural area where there was a shortage of oral vitamin B treatment (combination of vitamin B1, B6 and B12) and because of general containment and the curfew in Tunisia the patient's family did not have been able to obtain this treatment.

**Figure 1:**
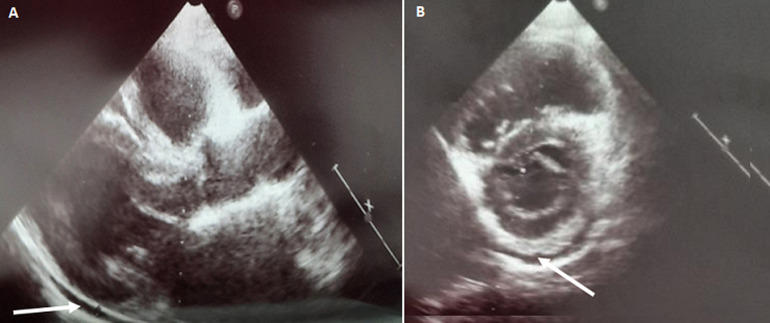
(A,B) pericardial effusion, in the cardiac ultrasound, in an adult patient with thiamine-responsive megaloblastic anaemia (TRMA) on discontinuation of oral thiamine-based treatment

**Investigations:** on admission, Body temperature and physical examination, as well as ECG, chest radiograph and urinalysis were normal. Blood tests revealed bicytopenia (haemoglobin 5.4 g/dl, mean corpuscular volume (MCV) 92 fL, haematocrit 23%, reticulocytes 30×10^9^/L (1%), thrombocytes 38×10^9^/L, leucocytes 6.2×10^9^/L) and negative C reactive protein. Serum ferritin level was 289 μg/L, folate concentration 45.4 nmol/L. There was no coagulopathy (D-dimer, prothrombin time, partial thromboplastin time and fibrinogen values were normal). Fasting and postprandial serum glucose levels were elevated (between 8 and 18.6 mmol/L without ketosis), as well as the HbA1c level (7.8%). To rule out the diagnosis of acute coronary syndrome in front of the chest pain presented by the patient, the ECG, repeated several times, was normal, two points of hypersensitive troponin was negative, a cardiac ultrasound had shown pericardial effusion of two millimeters circumferential (Figure 1 (A, B)), good left ventricle function with 74% of ejection fraction, without ischemic signs and without valve diseases, right cavities were normal.

**Treatment:** parenteral thiamine supplementation (100 mg a day during the first 10 days) was started on day 1, followed by oral supplementation (300 mg one time a day).

**Figure 2:**
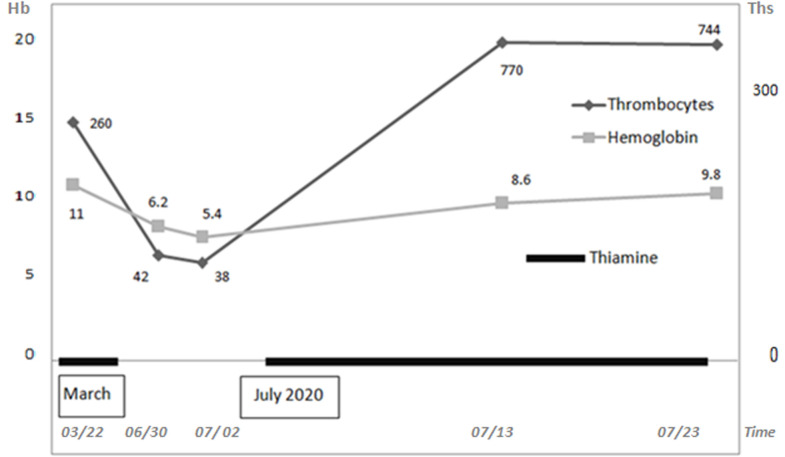
evolution of thrombocyte and haemoglobin counts over time. Thiamine supplementation was stopped from 30^th^ March to 3^rd^ July included. Hemoglobin (Hb) level is expressed in gram per litre (g/L). Thrombocyte (ths) counts are expressed in giga per litre (cells ×10^9^/L)

**Outcome and follow-up:** within 10 days of thiamine supplementation, reticulocytes rose to 200 ×10^9^/L, haemoglobin level to 9.8 g/dL, and platelets to 740 ×10^9^/L. Probably caused by the rapid onset of anemia, dyspnea and chest pain disappeared once anemia was partially corrected. The insulin requirements did not change. The patient was discharged on day 7 and oral thiamine supplementation (300 mg daily) was continued. Three weeks after initiation of treatment, the blood cell counts returned to normal values (Figure 2).

## Discussion

Thiamine-responsive megaloblastic anemia syndrome (TRMA) is also known as Roger's syndrome named after Roger *et al*. credited for being the pioneer group to report the disorder in 1969 [1]. TRMA is an uncommon genetic disorder caused by mutation of the SLC19A2 gene responsible for the production a transport protein called thiamine transporter [2]. The result is an abnormal thiamine transportation and thiamine deficiency in the cells. The anemia is corrected with high doses of thiamine and recurred when thiamine is withdrawn. In addition, supplement of high dose of thiamine could improve in the clinical symptoms of the disease including a reduction in the need for exogenous insulin in these patients [3]. The medical history and initial presentation of our patient were similar to other cases reports with the classic triad of manifestations: megaloblastic anaemia, diabetes mellitus and sensorineural deafness, diagnosis made during childhood. Most cases have been reported in consanguineous families [4] our patient did not have a family history of consanguinity. Most cases of TRMA have been reported in children population, with anaemia. A case of pancytopenia have been reported in a 25-year-old woman [5], the clinical presentation of this patient was similar to that of our patient. Pancytopenia appeared 5 weeks after the discontinuation of thiamine supplementation, probably because of depletion of thiamine reserves and complete recovery of haematopoiesis was obtained within 3 weeks once thiamine supplementation was reintroduced [5]. On the other hand, pericardial effusion, associated directly with decompensation of TRMA syndrome, has not been reported. It can be attributed most likely to vitamin B1 deficiency, this has been described in patients with beriberi syndrome [6,7]. The hypotheses on the etiology of bicytopenia in our patient was the discontinuation of thiamine supplementation, iron and vitamin deficiencies and an acute infection. In view of the complete recovery of haematopoiesis obtained within 3 weeks once thiamine supplementation was reintroduced, the etiology of the haematological disorders was attributed to the discontinuation of thiamine supplementation because of the containment during COVID-19 pandemic.

After the declaration of the COVID-19 pandemic by the WHO (World Health Association) on March 11, 2020 [8], general containment in Tunisia began on March 22, 2020 and lasted 10 weeks. Outings prohibited except extreme emergency and by respecting the barrier measures. Moving between governorates was forbidden during this period. This period was hardly lived over the world and recent studies have shown that SARS-COV 2 infection containment measures have substantial short- and long-term consequences [9,10]; social distancing and quarantine restrictions will diminish physical activity and increase other unhealthy lifestyles, also reduce treatment adherence thus increasing rare and chronic diseases risk factors and worsening clinical symptoms [9,10]. The discontinuation of oral thiamine-based treatment in our case can be explained by the non-availability of treatment and the difficulties in accessing care during the containment imposed by the COVID-19 pandemic. Depressive behavior cannot be eliminated in the face of the psychological impact of this unprecedented global crisis, which has been suggested by several studies [11,12].

## Conclusion

General containment, imposed by the COVID-19 pandemic, was at the origin of an anarchy in the medical follow-up and the management of several uncommon diseases such as the Thiamine-responsive megaloblastic anemia. This syndrome is a rare genetic disorder characterized by megaloblastic anaemia, diabetes mellitus and sensorineural deafness. Lifelong daily oral thiamine supplementation is necessary. Adult patients may present severe hematological manifestations in case of thiamine deficiency. This case report illustrated the isolation and silent suffering of patients with rare diseases during this particular situation of COVID-19 pandemic and has shown the universal new need for virtual communication especially for caregiver who must adopt telehealth technologys to support their patients.
